# The cost-effectiveness of family/family-based therapy for treatment of externalizing disorders, substance use disorders and delinquency: a systematic review

**DOI:** 10.1186/s12888-016-0949-8

**Published:** 2016-07-13

**Authors:** Maartje Goorden, Saskia J. Schawo, Clazien A.M. Bouwmans-Frijters, Evelien van der Schee, Vincent M. Hendriks, Leona Hakkaart-van Roijen

**Affiliations:** Institute for Medical Technology Assessment & Institute of Health Policy & Management, Erasmus University Rotterdam, P.O. Box 1738, 3000 DR Rotterdam, The Netherlands; Brijder Addiction Treatment, Parnassia Group, Parnassia Addiction Research Centre (PARC), P.O. Box 53002, 2505 AA The Hague, The Netherlands; Department of Child and Adolescent Psychiatry, Curium, Leiden University Medical Center, Leiden University, P.O. Box 37, 2300 AA Leiden, The Netherlands

**Keywords:** Systematic review, Cost-effectiveness, Family/family-based therapy, Delinquency, Substance use disorders

## Abstract

**Background:**

Family therapy and family-based treatment has been commonly applied in children and adolescents in mental health care and has been proven to be effective. There is an increased interest in economic evaluations of these, often expensive, interventions. The aim of this systematic review is to summarize and evaluate the evidence on cost-effectiveness of family/family-based therapy for externalizing disorders, substance use disorders and delinquency.

**Methods:**

A systematic literature search was performed in PubMed, Education Resource information Centre (ERIC), Psycinfo and Cochrane reviews including studies conducted after 1990 and before the first of August of 2013. Full economic evaluations investigating family/family-based interventions for adolescents between 10 and 20 years treated for substance use disorders, delinquency or externalizing disorders were included.

**Results:**

Seven hundred thirty-one articles met the search criteria and 51 studies were initially selected. The final selection resulted in the inclusion of 11 studies. The quality of these studies was assessed. Within the identified studies, there was great variation in the specific type of family/family-based interventions and disorders. According to the outcomes of the checklists, the overall quality of the economic evaluations was low. Results varied by study. Due to the variations in setting, design and outcome it was not feasible to pool results using a meta-analysis.

**Conclusions:**

The quality of the identified economic evaluations of family/family-based therapy for treatment of externalizing disorders, adolescent substance use disorders and delinquency was insufficient to determine the cost-effectiveness. Although commonly applied, family/family-based therapy is costly and more research of higher quality is needed.

## Background

Family therapy and family-based treatment is considered an evidence-based practice treatment for children and adolescents with externalizing disorders, symptoms of delinquency and/or substance use disorder [1, 2]. Familial and extra-familial systems are known to influence the individual [3–7], and therefore family/family-based therapy is not only aimed at the individual youth but also at systems surrounding the individual. For instance, delinquency and substance abuse in adolescents have been shown to be influenced by family factors, like parenting style and attachment [3–7]. In addition, a recent review indicated that problems within the extra-familial system, like delinquent peers, problems with bonding at school and in the neighborhood are risk factors for delinquency and problem drinking [7]. As the individual, familial and extrafamilial systems are interconnected, family/family-based therapy not only positively affects the adolescent but also the family (family cohesion) and the extra-familial systems [8].

For the purpose of the present paper, family therapy and family-based treatment is broadly defined as treatments in which primarily family members and/or members of the families’ wider networks are involved in the treatment process of resolving problems for young people [9] as opposed to treatments that mainly or solely focus on the individual youth, or treatments that do not focus on youths’ problem behavior, like marital therapy.

Well-known forms of family/family-based treatments are Multisystemic therapy (MST) [10], Functional Family Therapy (FFT) [11] and Multidimensional Family therapy (MDFT) [12]. Although there is a large overlap between these types of therapies, there are also some differences [13]. For instance, in FFT and MST there is more focus on antisocial behavior. However, the degree of severity of the disorder is often higher in MST compared to FFT. More details of these differences are described in Appendix 1.

Recently, Von Sydow et al. [1] systematically reviewed studies on the effectiveness of family/family-based therapy for the treatment of children and adolescents who have externalizing disorders. Their study included disorders like substance abuse, attention deficit hyperactivity disorder, conduct disorder and symptoms of delinquency. They concluded that there is sound evidence that family/family-based therapy is effective with particularly large effect sizes for delinquency and substance abuse measures. However, in the meta analyses that were included in Von Sydow’s systematic review, more cautious conclusions regarding the effectiveness of systemic therapy were drawn.

Current health care policy in the Netherlands and elsewhere places emphasis on the provision of effective mental health services in a cost effective way. Family/family-based interventions are intensive as they consist of a relatively high number of sessions per week and subsequently are relatively expensive [14–16]. Therefore, there is a need for economic evaluations to assess whether additional effects gained through family/family-based therapy in comparison to alternative treatments – if observed – justify the additional costs. Morgan et al. [17] described eight studies, analyzing the cost-effectiveness of family-based treatments for substance abusing adults and adolescents and concluded that some of these treatments could be considered as cost-effective. However, family based therapies like marital therapy, were also included in this study. In addition, the literature search in this study was not systematically conducted and was only considering patients with substance use disorders. To our knowledge, no systematic review of economic evaluations of family/family-based therapy in externalizing, delinquent or substance-abusing adolescents has yet been performed.

Hence, this paper presents a systematic review of economic evaluations of systemic interventions in adolescents with externalizing disorders, substance abuse or delinquency. The aim of the present study was to assess the evidence on cost-effectiveness of family/family-based therapy for adolescents with externalizing disorders, substance use disorders or delinquency, and to evaluate the quality of the existing studies, and the generalizability of the study findings.

## Methods

The review was performed according to the Cochrane handbook for systematic reviews of interventions [18] and adopted the Preferred Reporting for Systematic reviews and Meta-Analyses (PRISMA) statement [19].

### Search strategy

A systematic literature search was performed in Pubmed, ERIC, Psycinfo and Cochrane reviews (including economic trials and clinical trials). These different search engines were used because of their high quality, coverage of large databases and their focus on economic trials.

Search terms encompassed the different types of systemic therapy (Functional Family Therapy, Multidimensional Family therapy, Multidimensional Foster Care, Multisystemic Therapy, Family Behavior Therapy and Brief Strategic Therapy) but also more general classifications (systemic therapy, substance abuse treatment, family based therapy, Family based intervention, Family system intervention, Family intervention program). These terms were searched for in title and abstract and were then combined with terms referring to economic evaluations searched for in title and abstract or a Medical Subject Headings (MeSH) term (economic evaluation, cost-effectiveness, cost-utility, cost benefit, cost analysis, cost measure) and in the title (costs). Costs were searched for only in the title, and not in the abstract, because the latter resulted in many irrelevant studies. This search term was included as we noticed that although in some studies both costs and effects were evaluated, the main focus of these studies was to evaluate the costs and a smaller part was referring to the effects. Consequently, when only terms referring to both the costs and effects were included, these studies would have been missed. The search term “Economic modeling” was not explicitly incorporated into the search strategy as the modeling should be part of a cost-effectiveness, cost utility, cost benefit or cost analysis (corresponding with our aim).

Abbreviations were also included. To improve our search, MeSH terms were used, see Appendix 2 for more details.

### Selection strategy

**Fig. 1:**
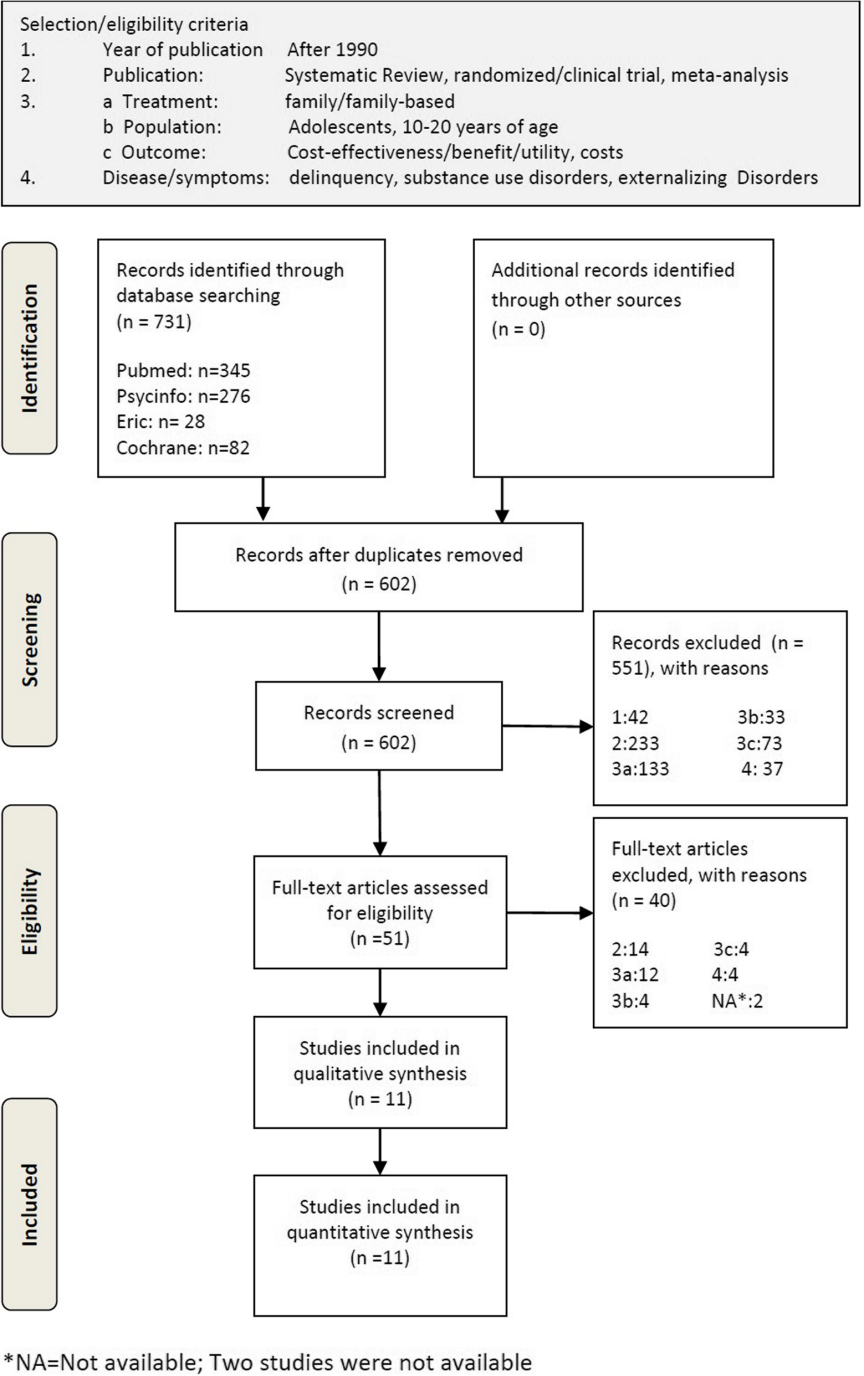
PRISMA flow diagram [19]

In Fig. 1 the selection criteria are described and numbered. The criteria were applied to the studies in chronological order and when a study was excluded based on a criterion the number as shown in Fig. 1 was noted. We considered studies from January 1990 until January 2016. The selected study types were clinical/randomized controlled trials (RCT), reviews, systematic reviews and meta-analyses. The treatment needed to consist of a family/family-based intervention, targeted at adolescents (10–20 years old) with a substance use disorder, externalizing disorder or delinquent behavior. The method needed to be a cost or cost- effectiveness/benefit/ utility analysis. When studies were assessed for eligibility based on their abstracts and it was likely that they only contained cost-outcomes and no effect-outcomes, they were also included. To determine the eligibility of the full text articles, the same selection criteria were used, except that accessibility of the study was a requirement (full text available) and studies that only contained costs-outcomes and no effect-outcomes were excluded. The selection of the articles was performed by two researchers independently. Differences in selections were discussed until consensus was reached.

### Data extraction and risk of bias

The quality of the studies was assessed with the British Medical Journal Checklist for authors and peer reviewers of economic submissions [20] and the Consensus on Health Economic Criteria (CHEC) list for assessment of methodological quality of economic evaluations [21] as recommended by the Cochrane reviews handbook [18]. We also consulted the critical appraisal of the studies by the NHS Economic Evaluation Database (NHS EED) structured abstract [18]. This is a database from Cochrane library consisting of structured abstracts of economic evaluations of health care interventions. Full economic evaluations were identified from a variety of sources and assessed according to a set of quality criteria. Subsequently, detailed structured abstracts were produced. In addition to the checklists, information about the economic perspective of the study (health care, societal etc.), design, country, follow-up, type of disorder, sample size, study dropout, age, gender, type, duration and intensity of intervention, time horizon, currency and price year, key features of sensitivity analyses and the included cost types were collected for the economic evaluation described in the studies. In accordance with the suggestions in the Cochrane handbook [18] five different biases of the individual studies were addressed: selection bias, performance bias, detection bias, attrition bias and reporting bias [18]. They were respectively addressed by assessing if patients were properly balanced at baseline, patients and therapists were blinded, outcome assessors were blinded, the amount of dropout in the studies and by reading the protocols of the studies.

## Results

A total of 731 articles met the search criteria. After removal of duplicates and a first selection based on the abstracts, 51 studies matched the inclusion criteria. After assessment for eligibility, 11 studies were selected (see Fig. 1).

### Characteristics of the studies

An overview of the characteristics of the studies, participants and the interventions is shown in Table 1. Ten of the eleven selected studies were published between 2003 and 2015 [22–31] and one study was published in 1996 [32]. Eight of the studies originated from the United States (USA) [22–24, 27, 29–32]. Remaining studies were initiated in Sweden [26], England [28] and Mexico [25]. All studies were (based upon) randomized controlled trials. Two pairs of studies [22, 24, 27, 29] were each based on one sample. Most of the studies compared a family/family-based intervention with care as usual [23, 26, 28, 30–32]. MST was the most researched intervention as it was investigated in eight studies [23, 24, 26–28, 30–32]. In the Study of Borduin et al. [31] Multisystemic Therapy for Problem Sexual Behavior (MST-PSB) was investigated. MST-PSB is an adaptation to MST aimed at the treatment of juvenile sexual offenders. A description of the (non- family/family-based) comparator interventions is shown in Table 2. The mean number of sessions of the family/family-based interventions was between 1 and 3 times a week and the mean duration of treatment was between 12 and 31 weeks. The average follow-up time was between 6 and 300 months (25 years); only four studies followed patients for more than 1 year [26, 28, 30, 31]. Two studies were outliers in respect to the time horizon they used (8 years and 25 years) [30, 31].

**Table 1:** Features of the studies, participants and the interventions

Study	Features study			Features participants									Features intervention			
	Coun-try	Follow-up (months)	Design	Disorder	Sample size		Completed study		Age		Sex (% male)		Intervention		Number of sessions per week	Treatment duration (weeks)
					I	C	I	C	I	C	I	C	I	C		
Schoenwald et al., 1996 [32]	USA	6 AT	RCT	SUD	59	59	NS		16		79		MST	CAU	2–3^b^	18–19
French et al., 2003 [22]	USA	12^a^	RCT	SUD									Trial 1:			
					102		564		16		81		MET/CBT5		0–1	6–7
					96				16		86		MET/CBT12		0–1	12–14
					102				16		84		FSN		1–2	12–14
													Trial 2:			
					100				16		79		MET/CBT5		0–1	6–7
					100				16		80		ACRA		1–2	12–14
					100				16		85		MDFT		1–2	12–14
Sheidow et al., 2004 [23]	USA	12 AT	RCT	PC	115		NS		13		67		MST	CAU	NS	16
Dennis et al., 2004 [29]	USA	12	RCT	SUD			564						Trial 1:			
					102				16				MET/CBT		0–1	6–7
					96				16				5		0–1	12–14
					102				16				MET/CBT		1–2	12–14
													12			
					100				16				FSN		0–1	6–7
					100				16				Trial 2:		1–2	12–14
					100				16				MET/CBT		1–2	12–14
													5			
													ACRA			
													MDFT			
McCollister et al., 2009 [24]	USA	12	RCT	SUD	38	42	NS		1	1	84	81	DC	FC	NS^1^	NS^1^
					38				5	5	84		DC + MST			
					43				15		4		DC + MST + CM			
									15							
French et al., 2008 [25]	MEX	7	RCT	SUD	30	30	114		16	1	80	83	FFT	group	NS^1^	NS^1^
					29				16	6	76		Joint			
					31				16		84		CBT			
Olsson, 2010 [26]	SW	24	RCT	CD	79	77	NS		15		61		MST	CAU	NS	12–20
Sheidow et al., 2012 [27]	USA	12	RCT	SUD	38	4	29	33	15		83		DC	FC	NS^1^	NS^1^
					38	2	29						DC + MST			
					43		37						DC + MST + CM			
Cary et al., 2013 [28]	ENG	30	RCT	DEL	56	52	46	45	15	15	83	82	MST+	CAU	3	20
													CAU			
Dopp et al. (2014) [30]	USA	300	RCT	DEL	92	84	70	56	15		69		MST	CAU	3–4	21
Borduin et al. (2015) [31]	USA	107	RCT	DEL	24	24	24	22	14		96		MST-PSB	CAU	3	31

*Legend*: *I* intervention, *C* comparator, *NS* not stated, *NS* ^*1*^ reference to non-accessible article, *NA* not applicable, *USA* United States of America, *SW* Sweden, *ENG* England, *MEX* Mexico, *SUD* substance use disorder, *CD* conduct disorder, *PC* psychiatric crisis, *MST* multisystemic therapy, *Joint* combination of individual and family therapy, *group* skill-focused psycho-education group intervention, *IT* individual treatment, *MST-PSB* MST for Problem Sexual Behavior, *CAU* care as usual, *FSN* family support network, *MDFT* multidimensional family treatment, *MET/CBT12* motivational enhancement treatment/cognitive behavior therapy, 12 sessions; *MET/CBT5* motivational enhancement treatment/cognitive behavior therapy, 5 sessions; *ACRA* adolescent community reinforcement approach, *DC* drug court with community services, *DC + MST* drug court with multisystemic therapy, *DC + MST + CM* drug court with mst and enhanced with a contingency management programs, *FFT* functional family therapy, *FC* family court with community services

^a^Cost data was only collected only during 3–9 months

^b^The intensity of the treatment was between 2 and 3 times a week; *AT* after treatment

**Table 2:** Descriptions of comparator interventions

FSN	Cognitive behavioral sessions and motivation treatment in combination with a family component.
MET/CBT5	Motivational component and a cognitive behavioral component, to enhance motivation to change drug abuse and to grow the skills to maintain and regulate abstinence
MET/CBT12	MET/CBT5+ 7 sessions of CBT are added to the therapy.
FC	Family court treatment with community services/Appearance court 2 times a year/ outpatient alcohol and drug abuse service from the local center of the state’s substance abuse commission
DC	Drug court treatment with community services/Appearance court 1 time a week/ outpatient alcohol and drug abuse service from the local center of the state’s substance abuse commission and monitoring drug abuse
CM	Frequent in home screens for drug use, voucher system contingent on clean screens, and drug refusal training.
ACRA	Identifying reinforces that are incompatible with the drug use and to strengthen those
CAU	Sheidow et al. [23]: admission to a psychiatric unit and aftercare
	Schoenwald et al. [32]: outpatient substance abuse services
	Olsson et al. [26]: Not described
	Cary at al. [28]: Youth Offending Team (YOT)
	Dopp et al. [30]; Individual Therapy (IT)
	Borduin et al. [31]: Cognitive behavioral group therapy and individual services (from local juvenile court)

*FSN* family support network, *MET/CBT5* motivational enhancement treatment/cognitive behavior therapy, 5 sessions, *MET/CBT12* motivational enhancement treatment/cognitive behavior therapy, 12 sessions; *ACRA* adolescent community reinforcement approach, *FC* family court with community services, *DC* drug court with community services, *CM* contingency management programs, *CAU* care as usual

Six studies were aimed at adolescents with substance use disorder [22, 24, 25, 27, 29, 32], one study investigated adolescents with a conduct disorder [26], one study adolescents at risk for continuing criminal activity [28],one study adolescents who had experienced a psychiatric crisis [23], another study adolescents who were serious juvenile offenders [30] and one study aimed to investigate juvenile sexual offenders [31]. The average sample size of the 9 studies (with separate samples) was 178 (SD = 163) with a variation between 48 and 600 patients. Follow-up attrition, when registered, was low (not more than 30 %). Average age at baseline was 15 (Standard Deviation (SD) = 1) years and between 61 and 96 % of the individuals were males.

Types of economic analyses included cost-effectiveness analyses [23, 25, 27, 29], cost-benefit analyses [22, 26, 30, 31] and cost offset analyses [28]. The difference between a cost-offset and a cost-benefit analysis is often not well-explained. A cost-offset analysis compares the monetary value of resource use with the monetary value of costs reduced by the intervention (usually health care costs). In contrast to a cost-benefit analysis which also focusses on other outcomes that are translated in monetary outcomes (like translating number of life years gained to a monetary value). In reality, cost-offset analysis is a partial cost-benefit analysis because it compares the cost of a program with the monetary value of a single outcome (i.e., avoided future health care costs). In two studies, the economic evaluation was not explicitly classified [24, 32].

### Outcomes of the studies

Details of the interventions and outcomes of our analyses are described in Tables 3 and 4. Costs were indexed until 2014.

**Table 3:** Studies that reported substance use disorders

Studies considering costs and effects of substance abuse						
Dennis (2004) [29]		Costs intervention and comparators (per episode of care per patient) (MET/CBT 5, MET/CBT 12, FSN, ACRA, MDFT) In trial 1 MET/CBT 5,MET/CBT 12 and FSN were compared. In trial 2 MET/CBT 5, ACRA and MDFT were compared. Costs were collected with a program (DATCAP) which yields estimates such as the total annual opportunity cost of treatment and the labor cost per client.			Difference cost	
					The difference in costs were not showed in this study. However, it was showed that the differences were significant.	
		MET/CBT 5 (trial 1): € 1,226 MET/CBT 12 (trial 1): € 1,305 FSN (trial 1): € 3,576	MET/CBT 5 (trial 2): €1,716 ACRA (trial 2): € 1,551 MDFT (trial 2): € 2,205			
		Effects intervention and comparators (per patient) (MET/CBT 5, MET/CBT 12, FSN, ACRA, MDFT)			Difference effects	
		Met CBT 5 (trial 1) Days of abstinence: 269 Recovery*: 28 % Met CBT 12 (trial 1) Days of abstinence: 256 Recovery: 17 % MET FSN (trial 1) Days of abstinence: 260 Recovery: 22 % *Recovery is defined as having no use or abuse dependence problems and living in the community	Met CBT 5 (trial 2) Days of abstinence: 251 Recovery: 23 % ACRA (trial 2) Days of abstinence: 265 Recovery: 34 % MDFT (trial 2) Days of abstinence: 257 Recovery: 19 %		The difference in effects were not showed in this study However it was showed that the difference was not significant.	
	Results	Cost per day of abstinence: Met CBT5 (trial 1): € 541 Met CBT 12: € 677 Met FSN: € 1,667 Costs per person in recovery Met CBT5 (trial 1): € 4,360 Met CBT 12: € 41,172 Met FSN: € 16,651			Cost per days of abstinence: MET/CBT5 (trial 2): € 991 ACRA: € 729 MDFT: € 1,143 Costs per person in recovery MET/CBT5 (trial 2): € 7,337 ACRA: € 4,913 MDFT: € 12,970	
French (2008) [25]		Costs intervention per patient (FFT, Joint and CBT) FFT: Treatment costs: € 1,817 Joint: treatment costs: € 2,847 CBT: Treatment costs: € 1.439	Costs comparator per patient (Group) Group: Treatment costs: € 990		Difference costs	
					The difference in costs were not showed in this study	
		Effects intervention per patient (FFT, Joint and CBT) FFT: % days marijuana use 4 months: 25.3 %of days marijuana use 7 months:39.8 YSR delinquency score 4 months: 8.2 YSR delinquency score 7 months:9.2 Joint % of days of marijuana use 4 months: 38.1 marijuana use 7 months:35.4 YSR delinquency score 4 months: 9.1 YSR delinquency score 7 months:8.5 CBT % of days marijuana use 4 months: 50.6 % of days marijuana use 7 months:51.8 YSR delinquency score 4 months: 10.2 YSR delinquency score 7 months:10.4	Effects comparator per patient (Group) Group % of days of marijuana use 4 months: 54.8 marijuana use 7 months: 40.7 YSR delinquency score 4 months: 9.5 lYSR delinuency score 7 months: 9.4		Difference effects with regression model:	
					FFT versus group: % days marijuana use after 4 months: −20.11* % days marijuana use after 7 months: 4.87 YSR delinquency score 4 months: −0.60 YSR delinquency score 7 months: 0.15 CBT versus group % days marijuana use after 4 months: 4.76 % days marijuana use after 7 months: 18.27 YSR delinquency score 4 months: 0.38 YSR delinquency score 7 months: 0.42 Joint versus group % days marijuana use after 4 months: −14.86 % days marijuana use after 7 months “-2.00 YSR delinquency score 4 months: −0.50 YSR delinquency score 7 months: −1.50	Joint versus group Delinquency score after 4 months: −0.50 Delinquency score after 7 months: −1.50
	Results	Group therapy was most cost-effective, none of the other therapies were significantly different in effect compared to group therapy. So the intervention with the lowest costs was considered to be most cost-effective.				
Sheidow (2012) [27]		Costs Intervention (DC, DC + MST, DC + MST + CM) Treatment costs DC: € 9,083 Treatment costs DC + MST: € 12,369 Treatment costs DC + MST + CM: € 12,859	Costs comparator (FC) Treatment costs FC: € 3,679		Difference costs:	
					The difference in costs were not shown in this study	
		Effects intervention (DC, DC + MST, DC + MST + CM) DC Marijuana use (days): −16.65 Polydrug use (days): 1.41 Alcohol use (days): 0.49 Heavy alcohol use (days): 0.86 SRD status offenses (incidents): −7.24 SRD Theft (incidents): −3.28 SRD crimes against persons (incidents): −2.69 DC ± MST Marijuana use (days): −30,17 Polydrug use (days): :-1.11 Alcohol use (days): 0.27 Heavy alcohol use (days): −0.45 SRD status offenses (incidents): −11.11 SRD Theft (incidents): −2.79 SRD crimes against persons (incidents): −3.90 DC ± MST ± CM Marijuana use (days): −27.86 Polydrug use (days): −6.76 Alcohol use (days): −7.56 Heavy alcohol use (days): −4.13 SRD status offenses (incidents): −10.38 SRD Theft (incidents): −3.19 SRD crimes against persons (incidents): −2.4	Effects comparator (FC) Marijuana use (days): −15,43 Polydrug use (days): 2.27 Alcohol use (days): 2.97 Heavy alcohol use (days): 0.76 SRD status offenses (incidents): 9.22 SRD Theft (incidents): −5.54 SRD crimes against persons (incidents): 0.49		Difference effects: The difference in effects were not showed in this study	
	Results	ACERS were calculated; average costs/ difference between mean incidents before and after treatment (negative means inefficient)				
			FC	DC	DC + MST	DC + MST + CM
		Marijuana use: Polydrug use: Alcohol use: Heavy alcohol use: SRD status offenses: SRD theft: SRD crimes against persons:	€ 238 (215–262) € −1,619 (−8,8839–5,601) € −,1,239 (−6,546–5,601) € −4,857 (−10,632–918) € −400 (−1,206–398) € 663 (428–899) € −7,588 (−10,667–4,510)	€ 545 (474–617) € −6,425 (−27,541–14,692) € −18,814 (−42,034–4,405) € −10,535 (−28,804–7,733) € 1,254 (1,132–1,376) € 2,773 (−2.441–7,987) € 3,377 (2,976–3,777)	€ 410 (377–442) € 11,209 (−3,757–26,175) € −44,838 (−61,014–28,662) € 27,592 (−14,636–69,821) € 1,114 (907–1,321) € 4,428 (−1,224–10,081) € 3,175 (236–6,123)	€ 461 (434–488) € 1,912 (1,624–2,182) € 1,699 (1,486–1,912) € 3,109 (1,708–4,511) € 1,239 (1,009–1,496) € 4,032 (1,204–6,859) € 5,346 (4,723–5,968)
Studies considering costs and benefits of substance abuse						
Schoenwald (1996) [32]		Costs interventions (MST) Mental health outpatient (total): €4,242 Mental health day treatment (total): € 5,423 Mental health residential treatment (total): €6,899 Psychiatric inpatient (total): €15,752 Psychiatric emergency room (total): €1,150 Substance abuse outpatient (total): € 2,001 Substance abuse residential treatment (total): €3,450 Substance abuse inpatient (total): € 16,098 Marine Institute day treatment (total): € 18,926 Marine Institute residential treatment (total): € 3,036 Treatment costs: € 266,516	Costs comparator (CAU) Mental health outpatient (total): € 19,075 Mental health day treatment (total): € 1,118 Mental health residential treatment (total): €0 Psychiatric inpatient (total): €18,513 Psychiatric emergency room (total): €3,450 Substance abuse outpatient (total): € 20,272 Substance abuse residential treatment (total): €43,695 Substance abuse inpatient (total): €93,771 Marine Institute day treatment (total): €28,618 Marine Institute residential treatment (total): €0		Benefits interventions Incarceration days: €65,427	Benefits CAU Incarceration days: €120,851
	Results	MST: Total costs (costs + benefit) with incarceration = €408,919 and the total costs (costs + benefit) with incarceration per youth = €6,930 CAU: Total costs (costs + benefit) with incarceration = € 335,845 and the costs (costs + benefit) per youth = €5,693. Difference in total between groups = €1,019				
French (2003) [22]		Costs interventions (MET/CBT 5, MET/CBT 12, FSN, ACRA, MDFT)			Benefits interventions (MET/CBT 5, MET/CBT 12, FSN, ACRA, MDFT)	
		Treatment costs were measured			Health service utilization; Outpatient clinic/doctor’s office visit Days bothered by health/medical problem Substance-absue treatment utilization; Days in detoxification program; Day in inpatient treatment program; Day in long-term residential program; Intensive outpatient program visits; Regular outpatient program visits Education and employment; Days missed at school or training; Personal income; Days stressful for parents Day missed of work or school by parent Criminal activity; Arrests; Day on probation; Days on parole; Days in prison/jail; Days in juvenile detention	
		Incremental arm: MET/CBT5:€ 1,226 MET/CBT12: € 1,305 FSN: € 3,576	Alternative arm: MET/CBT5: € 1,716 ACRA: € 1,551 MDFT: € 2,216		Incremental arm: MET/CBT5 Baseline € 2,553 3 months: € 2,133 6 months: € 1,671 9 months: € 945 12 months: € 1,217 MET/CBT12 Baseline: € 2,179 3 months: € 2,433 6 months: € 828 9 months: € 1,431 12 months: € 687 FSN: Baseline: € 2,552 3 months: € 4,525 6 months: € 1,783 9 months: € 1,205 12 months: € 1,726	Alternative arm: MET/CBT5 Baseline € 2,694 3 months: € 3,587 6 months: € 2,213 9 months: € 2,275 12 months: € 1,907 ACRA Baseline: € 2,506 3 months: € 3,691 6 months: € 1,748 9 months: € 3,113 12 months: € 3,237 MDFT: Baseline: € 2,019 3 months: € 3,938 6 months: € 1,467 9 months: € 2,573 12 months: € 2,098
	Results	Net economic benefits (benefits + costs) relative to baseline: 3 different models were administred; Model 1: only time dummies for each of the follow-up periods (as treatment conditions were not included, we did not show the results. Model 2: time dummies and indicator variables for treatment condition. Model 3: time and treatment variables withan indicator variable for site. The last specification added numerous demographic and environmental controls.				
		MET/CBT12: Model 2: € −198 (349) Model 3: € −171 (346) Model 4: € −340 (334) FSN: Model 2: € 607* (343) Model 3: € 653 (340) Model 4: € 250 (333) *p < 0.1			Acra: Model 2: € 369 (436) Model 3: € 530 (430) Model 4: € 554 (405) MDFT Model 2: −€ 61 (441) Model 3: € 128 (436) Model 4: € 100 (530)	
McCollister (2009) [24]		Costs interventions (DC, DC/MST, DC)	Costs comparators (FC)		Benefits interventions (DC, DC/MST, DC)	Benefits comparators (FC)
		Treatment costs	Treatment costs		Criminal activity costs according to Self-reported criminal activity (SRD): DC: € 28.601 (94.314) DC/MST: €65.640 (240.559) DC/MST/CM: €80.222 (336.461)	Self-reported criminal activity (SRD): FC: € 206.045 (545.581)
		DC: €8,156 DC/MST: €11,547 DC/MST/CM: € 11,547	FC: €3,304			
	Results	After 12 months, total costs relative to FC with multivariate model (intervention costs not incorporated): DC: € -124,877 (−84,107) DC/MST: €-117,918 (−82,570) DC/MST/CM: €140,274 (/79.066)* All DC conditions generated reduction in crime costs, greater than average costs of treatment.				

Currency and price year: Sheidow (2004).USD, 1997; Dennis (2004).USD, 1999; French 2008.USD, 1998; Sheidow (2012).USD 2004. When a price year was not stated it was estimated by taking the mean year of the study duration or when not available subtracting 1 from the year of publication of the study

*MST* multisystemic therapy, *Joint* combination of individual and family therapy, *group* skill-focused psycho-education group intervention, *CAU* care as usual, *FSN* family support network, *MDFT* multidimensional family treatment, *MET/CBT12* motivational enhancement treatment/cognitive behavior therapy, 12 sessions, *MET/CBT5* motivational enhancement treatment/cognitive behavior therapy, 5 sessions, *Acra* adolescent community reinforcement approach, *DC* drug court with community services, *DC* + *MST* drug court with multisystemic therapy; *DC* + *MST* + *CM* drug court with MST and enhanced with a contingency management programs, *FFT* functional family therapy, *FC* family court with community services, *ACERS* average cost-effectiveness ratios

**Table 4:** Studies considering externalizing disorders and delinquency

Studies considering both costs and effects					
Sheidow (2004) [23]		Costs intervention (MST) Medicaid (government insurance program) costs (inpatient, Outpatient, Pharmacy, other costs), Other treatment costs paid for by study MST Medicaid costs: 0-end treatment (4 months): €9,311 (±7,755) Medicaid costs: End treatment-12 months: €13,237 (±15,144) Other treatment costs paid for by study: €11,617	Costs comparator (CAU) Medicaid (government insurance program) costs (inpatient, Outpatient, Pharmacy, other costs), Other treatment costs paid for by study CAU Medicaid costs: 0-end treatment (4 months): €13,255 (±5,762) Medicaid costs: End treatment-12 months: €15,207 (±18,485) Other treatment costs paid for by study: €0	Difference costs (Costs_CAU_-Costs_MST_) (after risk adjusted model):	
				0-end treatment (total costs): End treatment- 12 months post-treatment (total costs):	-€ 1,828 -€452 (SE = 14)
		Effects intervention CBCL: Externalizing scores, internalizing scores: GSI: Global severity index are measures The main effects were not showed in this study but only differences over time were presented.	Effects comparator CBCL: Externalizing scores, internalizing scores: GSI: Global severity index The main effects were not showed in this study but only differences over time were presented.	Difference effects (Effects_CAU_-Effects_MST_) (after risk adjusted model):	
				0-end treatment: end treatment- 12 months post-treatment:	Externalizing:-14.75 (SE = 8.37) Internalizing:-14.19 (SE = 9.26) Global severity index: −0.03 (SE = 0.497) Externalizing:3.29 (SE = 9.97) Internalizing:-6.18 (SE = 9.67) Global severity index: −0.37 (SE = 0.428)
	Results	ICER: 1 point improvement in externalizing scores for usual care was associated with a cost of €1,561. 1 point improvement in externalizing scores for MST was associated with a costs of €404. After 12 months both treatments have comparable costs and externalizing scores.			
Studies considering costs and benefits					
Olsson^4^ (2010) [26]		Costs intervention (MST) Treatment costs: € 10.789 Travel: € 53 (133)	Costs comparator (CAU) Travel: €151 (225)	Benefits intervention (MST) Psychosocial and behavioral effects: − Social services (placement): € 31.947 (€65.869) Social services (nonplacement): € 8.557 (19.459) National board of institutional care (rebate): € 3.009 (11.014) National board of institutional care (placements): € 3.593 (31.937) Wider societal costs and benefit: set to zero Psychosocial and behavioral effects: set to zero	Benefits comparator (CAU) Program effects Social services (Placement): € 36.707 (73.407) Social services (nonplacement): € 14.914 (15.405) National board of institutional care (rebate): € 2.375 (9.949) National board of institutional care (placements): 0 (0) SEK Wider societal costs and benefit: set to zero
	Results	The net loss to society after two years is € 4.555			
Cary (2013) [28]		Costs interventions (MST + YOT) Treatment costs:€ 3.013 (1.940) Social worker: € 733 (446) Reparation worker: € 100 (131) Drugs worker: € 54 (74) Connexions worker: € 33 (69) Parenting worker: € 36 (137) Group worker: € 17 (34) Psychologist: € 17 (67) Other appointments:€ 20 (59)	Costs comparator (YOT) Social worker: € 1.023 (779) Reparation worker: € 83 (14) Drugs worker: € 78 (152) Connexions worker: € 18 (61) Parenting worker: € 90 (182) Group worker: € 22 (44) Psychologist: € 30 (91) Other appointments: € 26 (95)	Benefits interventions (MST + YOT)	Benefits comparator (YOT)
				Offending behavior (Young offender information system):	
				€ 12,397 (18 472)	€ 15,409 (24,013)
	Results	Difference (Costs + benefits) between treatments € 1.612 (95 % C.I-€ 7.699-€ to 10.924) In the cost-effectiveness plane, we see, there is 63 % probability that the net benefit of MST + Yot is positive in favor of the MST + YOT group.			
Dopp (2014) [30]		Costs interventions (MST) Costs per patient: € 9,756	Costs comparator (CAU) Costs per patient: € 1,843	Benefits intervention (MST)	Benefits comparator (IT)
				Benefits for taxpayer Murder: € 0 Sexual offenses: € 922 Robbery: € 188 Assault: € 1.156 Property: € 2.395 Drug: € 916 Theft: € 131 Stolen property: € 24 Fraud: € 259 Assault: € 236 Drug: € 777 TOTAL: € 7.007	Benefits for taxpayer Murder: € 0 Sexual offenses: € 602 Robbery: € 308 Assault: € 1.697 Property: € 1.899 Drug: € 1.334 Theft: € 188 Stolen property: € 53 Fraud: € 224 Assault: € 294 Drug: € 598 TOTAL: € 7.197
	Results	Crime victim avoided expenses Murder/manslaughter Tangible: € 6.125 Intangible: € 11.365 Sexual Tangible: € 259 Intangible: € 3.439 Robbery Tangible: € 575 Intangible: € 1.422 Assault Tangible: € 539 Intangible: € 2.926 Property Tangible: € 3.914 Intangible: € 0 Drug Tangible: € 0 Intangible: € 0 TOTAL Tangible: € 11.412 Intangible: € 19.151	Net present values and benefit-cost ratios Net present value Referred youths Taxpayer: € 2.348 Crime victim tangible: € 2.389 Crime victim intangible € 9.375 Cumulative: € 29.939 Siblings: Taxpayer: € 674 Crime victim tangible: € 2.702 Crime victim intangible: € 4.533 Cumulative: € 6.561 Sibling pairs Taxpayer: € 1.399 Crime victim tangible: € 3.499 Crime victim intangible: € 11.238 Cumulative*: € 31.962 *: Includes the incremental costs of MST over IT Benefit cost ratio Referred youths Taxpayer: 1.3 Crime victim tangible: 1.3 Crime victim intangible 2.19 Cumulative: 4.78 Siblings: Taxpayer: - Crime victim tangible: - Crime victim intangible: - Cumulative: - Sibling pairs Taxpayer: 1.18 Crime victim tangible: 1.44 Crime victim intangible: 2.42 Cumulative*: 5.04 *: Includes the incremental costs of MST over CAU	Sensitivity analysis Max (plausible) values Crime victim intangible benefits: € 48.087 Sibling juvenile arrest rates: € 30.74 Discount rates: € 24.063 Min (plausible) values Crime victim intangible benefits: € 17.561 Sibling juvenile arrest rates:- Discount rates: € 36.704	
Borduin (2015) [31]		Costs interventions (MST-PSB) Costs per patient: € 10,566	Costs comparator (CAU) Costs per patient: €4,610	Benefits intervention (MST-PSB) Benefits for taxpayer Murder: € 0 Sexual offenses: € 6.419 Robbery: € 2.189 Assault: € 0 Property: € 2.831 Drug: € 1.899 Theft: € 180 Stolen property€ 0 Fraud: € 91 Assault: € 250 Drug: € 512 TOTAL: € 14.371	Benefits comparator (CAU) Benefits for taxpayer Murder: € 0 Sexual offenses: € 15.756 Robbery: € 0 Assault: € 2.194 Property: € 3.790 Drug: € 518 Theft: € 65 Stolen property: € 39 Fraud: € 75 Assault: € 289 Drug: € 112 TOTAL: € 22.839
		Crime victim avoided expenses Murder/manslaughter Tangible: € 41.048 Intangible: € 76.169 Sexual Tangible: € 1.739 Intangible: € 23.044 Robbery Tangible: € 3.850 Intangible: € 9.529 Assault Tangible: € 3.612 Intangible: € 19.611 Property Tangible: € 26.244 Intangible: € 0 TOTAL Tangible: € 76.494 Intangible: € 128.353	Net present values and benefit-cost ratios Net present value Referred youths Taxpayer: € € 79.891 Crime victim tangible: € 70.538 Crime victim intangible € 122.397 Cumulative*: € 284.739 Siblings: Benefit cost ratio Referred youths Taxpayer: 14.41 Crime victim tangible: 12.84 Crime victim intangible 21.55 Cumulative: 48.81 *: Includes the incremental costs of MST over CAU	Sensitivity analysis Max (plausible) values Crime victim intangible benefits: € 387.085 Discount rates: € 239.009 Posttreatment arrest rates: € 478.277 Min (plausible) values Crime victim intangible benefits : € 188.217 Discount rates: € 311.107 Posttreatment arrest rates: € 91.673	

Currency and price year: Schoenwald 1996.USD, 1996; French 2003. United States Dollar (USD), 1999; Mc Collister (2009). USD,2008; Olsson (2010) Swedish krona (SEK), 2007; Cary (2013). Pounds, 2008; Dopp (2014) USD, 2012; Borduin (2015) USD, 2013. When a price year was not stated it was estimated by taking the mean year of the study duration or when not available subtracting 1 from the year of publication of the study. For Schoenwald et al. (2006), 1996 was taken as prices year although the study was also published in 1996. This was because they already published their first study in 1996 (preliminary findings) and subsequently probably the current study was conducted in 1996

*MST* multisystemic therapy, *Joint* combination of individual and family therapy, *group* skill-focused psycho-education group intervention, *CAU* care as usual, *FSN* family support network, *MDFT* multidimensional family treatment, *MET/CBT12* motivational enhancement treatment/cognitive behavior therapy, 12 sessions, *MET/CBT5* motivational enhancement treatment/cognitive behavior therapy, 5 sessions, *ACRA* adolescent community reinforcement approach, *DC* drug court with community services, *DC + MST* drug court with multisystemic therapy; *DC + MST + CM* drug court with MST and enhanced with a contingency management programs, *FFT* functional family therapy, *FC* family court with community services, *MST-PSB* MST for sexual behaviors; *ICER* incremental cost-effectiveness ratio

#### Substance abuse

Six studies were identified which included adolescents that were treated for substance abuse [22, 24, 25, 27, 29, 32]. Three of these studies considered costs and effects [25, 27, 29] and three considered both costs and benefits [22, 24, 32].

##### Studies considering costs and effects

In the study of French et al. [25] FFT was shown to be more cost-effective than a skill-focused psycho-education group intervention for treating substance use disorders and delinquency after the first 4 months. After 12 months no such effect was observed. Therefore, after 12 months the cost-effectiveness analysis reduced to a simple cost minimization analysis. As only treatment costs were considered (narrow perspective), the intervention with the lowest intervention costs, in this case group therapy, was considered to be economically beneficial. In another study, Dennis et al. [29] computed cost-effectiveness ratios and these ratios indicated that overall, the most cost-effective interventions were Motivational Enhancement Treatment/ Cognitive Behavior Therapy, 5 sessions (MET/CBT5) and Motivational enhancement treatment/ Cognitive Behavior Therapy, 12 sessions (MET/CBT12) when compared to Family Support Network (FSN) in Trial 1 and Adolescent Community Reinforcement Approach (ACRA) and MET/CBT5 when compared to MDFT in Trial 2. Sheidow et al. [27], computed Average Cost-Effectiveness Ratios (ACERS). ACERS only incorporate the pre-post treatment effect of one single treatment so treatments are not directly compared. Although this study showed that Drug Court with community services (DC) was more cost effective compared to FC regarding substance use disorders and that the addition of multi-systemic therapy (MST) resulted in an economically more beneficial treatment, the treatments were not directly compared [27].

##### Studies considering costs and benefits

Three of the studies that considered adolescents with substance use disorders, considered costs and benefits [22, 24, 32]. The study of French et al. [22] indicated that MET/CBT-5, MET/CBT-12 and FSN generated significant economic benefits to society for substance abusing adolescents, MDFT and ACRA did not generate these benefits. MCcollister et al. [24] showed that the savings in costs offset the treatment costs of DC, especially for DC/MST/CM, in juvenile drug court participants when compared to FC (Family court with community services). Schoenwald [32] showed that the monetary benefits of MST compared to CAU for substance use disorder almost offset the higher costs of MDFT. Over time the difference between benefits and costs may be reduced to a complete offset.

#### Delinquency/externalizing disorders

Five studies considered adolescents with delinquency or externalizing disorders; the study of Sheidow et al. [23], Olsson [26], Cary et al. [28], Dopp et al. [30] and Borduin et al. [31] respectively included patients with a psychiatric crisis, patients with a conduct disorder,delinquent adolescents, serious juvenile offenders and juvenile sexual offenders. One study, Sheidow et al. [23], considered both costs and effects and four studies [26, 28, 30, 31] considered both costs and benefits.

##### Studies considering costs and effects

In the study of Sheidow et al. [23], MST was effective in the short term (4 months) in terms of externalizing behavior compared to care as usual for patients with psychiatric emergencies. But MST appeared equally effective on the cost measure over the long term (12 months).

##### Studies considering costs and benefits

Olsson [26] showed that for adolescents with conduct disorder MST’s benefits did not offset the costs and that MST was subsequently associated with a net loss to society. The study of Cary et al. [28] showed that MST in combination with CAU has a scope to generate cost savings when compared to providing CAU alone. The cost-benefit study of Dopp et al. [30] indicated that MST, when delivered to serious juvenile offenders, produces economic benefits well into adulthood. Borduin et al. [31] showed that when juvenile sexual offenders are treated with MST-PSB; this treatment can produce lasting economic benefits.

### Quality of the studies

Only for one study [23] commentary was available from the NHS-EED. We compared the commentary on the study with our quality assessment checklists to evaluate if all issues were addressed. The quality of the studies was not only assessed for the 7 unique studies but for the 9 studies. The argument for including all studies was to differentiate between methods (e.g. analysis), display of results and discussion even though they were based on the same study. The quality assessed with the BMJ checklist was between 52 and 86 % (Table 5). The quality assessed with the CHEC list was between 50 and 79 % (Table 5). Up to date, there are no thresholds (minimum number of criteria satisfied) for these checklists to determine the difference between bad and good quality economic evaluations [18]. Overall, the outcomes on the checklists matched although quality assessed with the CHEC list was consequently lower. The largest difference in quality percentages was 20 %. All studies clearly stated their primary outcome measures. Most studies did not report all relevant costs and effects.

**Table 5:** Assessments of the quality of the studies with the Drummond checklist and the CHEC list

British Medical Journal Checklist	1^a^	2^a^	3^a^	4^a^	5^a^	6^a^	7^a^	8^a^	9^a^	10^a^	11^a^
1. The research question is stated.	-	-	✓	✓	✓	✓	✓	-	✓	✓	✓
2. The economic importance of the research question is stated.	✓	-	✓	✓	-	✓	✓	✓	✓	✓	✓
3. The viewpoint(s) of the analysis are clearly stated and justified.	-	✓	-	✓	-	-	✓	✓	-	-	-
4. The rationale for choosing alternative programmes or interventions compared is stated.	✓	-	-	-	-	-	✓	-	✓	-	-
5. The alternatives being compared are clearly described	✓	✓	✓	✓	-	-	-	-	✓	✓	✓
6. The form of economic evaluation used is stated.	-	✓	✓	✓	-	✓	✓	✓	✓	✓	✓
7. The choice of form of economic evaluation is justified in relation to the questions addressed.	NC	✓	✓	✓	-	✓	✓	✓	✓	✓	✓
8. The source(s) of effectiveness estimates used are stated.	✓	✓	✓	✓	✓	✓	✓	✓	✓	✓	✓
9. Details of the design and results of effectiveness study are given (if based on a single study).	✓	NA	✓	✓	✓	✓	✓	✓	-	✓	✓
10. Details of the methods of synthesis or meta-analysis of estimates are given (if based on a synthesis of a number of effectiveness studies).	NA	NA	NA	NA	NA	NA	NA	NA	NA	NA	NA
11. The primary outcome measure(s) for the economic evaluation are clearly stated.	✓	✓	✓	✓	✓	✓	✓	✓	✓	✓	✓
12. Methods to value benefits are stated.	✓	✓	NA	✓	✓	NA	✓	NA	✓	✓	✓
13. Details of the subjects from whom valuations were obtained were given.	✓	✓	✓	✓	✓	✓	✓	✓	✓	✓	✓
14. Productivity changes (if included) are reported separately.	NA	✓	NA	NA	NA	NA	NA	NA	NA	-	-
15. The relevance of productivity changes to the study question is discussed.	-	-	-	-	-	-	✓	-	-	-	-
16. Quantities of resource use are reported separately from their unit costs.	✓	✓	-	-	-	-	-	-	✓	✓	✓
17. Methods for the estimation of quantities and unit costs are described.	-	-	-	-	✓	-	✓	✓	✓	✓	✓
18. Currency and price data are recorded.	✓	✓	-	✓	-	-	-	✓	✓	✓	✓
19. Details of currency of price adjustments for inflation or currency conversion are given.	✓	✓	-	-	-	-	✓	-	✓	✓	✓
20. Details of any model used are given	NA	✓	✓	✓	✓	✓	NA	NA	✓	NA	NA
21. The choice of model used and the key parameters on which it is based are justified.	NA	-	-	✓	-	-	NA	NA	-	NA	NA
22. Time horizon of costs and benefits is stated.	✓	✓	✓	✓	✓	✓	✓	✓	✓	✓	✓
23. The discount rate(s) is stated.	NA	NA	NA	NA	NA	NA	✓	NA	✓	✓	✓
24. The choice of discount rate(s) is justified.	NA	NA	NA	NA	NA	NA	✓	NA	✓	✓	✓
25. An explanation is given if costs and benefits are not discounted.	NA	NA	NA	NA	NA	NA	NA	NA	NA	NA	NA
26. Details of statistical tests and confidence intervals are given for stochastic data.	-	-	✓	-	✓	-	✓	✓	✓	-	-
27. The approach to sensitivity analysis is given.	✓	-	✓	-	-	-	✓	NC	✓	✓	✓
28. The choice of variables for sensitivity analysis is justified.	✓	NA	NA	NA	NA	NA	✓	NA	✓	✓	✓
29. The ranges over which the variables are varied are justified.	NC	NA	NA	NA	NA	NA	✓	NA	✓	✓	✓
30. Relevant alternatives are compared.	✓	NC	-	NC	✓	NS	✓	✓	✓	✓	✓
31. Incremental analysis is reported.	✓	✓	-	✓	✓	-	✓	-	✓	✓	✓
32. Major outcomes are presented in a disaggregated as well as aggregated form	✓	✓	✓	-	✓	✓	✓	✓	✓	✓	✓
33. The answer to the study question is given.	✓	NC	✓	✓	✓	✓	✓	✓	✓	✓	✓
34. Conclusions follow from the data reported.	✓	✓	✓	✓	✓	✓	✓	✓	✓	-	-
35. Conclusions are accompanied by the appropriate caveats.	-	-	✓	✓	-	✓	✓	✓	-	✓	-
Total score British medical journal checklist	68 %	61 %	63 %	68 %	54 %	52 %	86 %	70 %	83 %	81 %	77 %
CHEC list											
1. Is the study population clearly described?	✓	✓	✓	✓	✓	✓	✓	✓	✓	✓	✓
2. Are competing alternatives clearly described?	✓	✓	✓	✓	-	-	-	-	✓	✓	✓
3. Is a well-defined research question posed in answerable form?	-	-	✓	✓	✓	✓	✓	-	✓	✓	✓
4. Is the economic study design appropriate to the stated objective?	✓	✓	✓	✓	✓	✓	✓	✓	✓	✓	✓
5. Is the chosen time horizon appropriate to include relevant costs and consequences?	NS	NS	✓	NS	NS	NS	✓	NS	NS	✓	✓
6. Is the actual perspective chosen appropriate?	-	✓	-	-	-	-	✓	-	-	-	-
7. Are all important and relevant costs for each alternative identified?	-	-	NS	-	-	-	-	-	-	✓	✓
8. Are all costs measured appropriately in physical units?	✓	✓	-	-	-	-	✓	-	✓	✓	✓
9. Are costs valued appropriately?	✓	✓	-	✓	✓	NS	✓	✓	✓	✓	✓
10. Are all important and relevant outcomes for each alternative identified?	-	-	✓	✓	-	✓	✓	✓	✓	✓	✓
11. Are all outcomes measured appropriately?	✓	✓	✓	✓	✓	✓	✓	✓	✓	✓	✓
12. Are outcomes valued appropriately?	-	✓	✓	✓	✓	✓	-	✓	✓	✓	✓
13. Is an incremental analysis of costs and outcomes of alternatives performed?	✓	✓	-	✓	✓	-	✓	-	✓	✓	✓
14. Are all future costs and outcomes discounted appropriately?	NA	NA	NA	NA	NA	NA	✓	NA	✓	✓	✓
15. Are all important variables, whose values are uncertain, appropriately subjected to sensitivity analysis?	✓	-	-	-	-	-	✓	-	✓	✓	✓
16. Do the conclusions follow from the data reported?	✓	✓	✓	✓	✓	✓	✓	✓	✓	-	-
17. Does the study discuss the generalizability of the results to other settings and patient/client groups?	-	-	✓	-	-	✓	✓	✓	-	-	-
18. Does the article indicate that there is no potential conflict of interest of study researcher(s) and funder(s)?	-	✓	-	-	-	-	-	-	-	-	-
19. Are ethical and distributional issues discussed appropriately?	✓	✓	✓	✓	✓	✓	✓	✓	✓	✓	✓
Total score CHEC^b^	56 %	67 %	61 %	61 %	50 %	50 %	79 %	50 %	79 %	74 %	74 %

*NS* not stated, *NA* not applicable, *NC* not clear

Explanation criteria checklist: British medical journal checklist: 1. A specific question is not necessary, as long as the goal of the research is clearly stated; 5. The competing alternatives may also be described in a different accessible paper from the RCT in more detail 10. The presentation of the results is clearly given and discussions of the study contain generalizability and comparison with other studies. CHEC list: 5: Chosen time horizon is appropriate when after a certain time no additional effects are attained

^a^Studies: Schoenwald et al., 1996; 2 French et al., 2003; 3 Sheidow et al., 2004; 4 Dennis et al., 2004; 5 McCollister et al., 2009; 6 French et al., 2008; 7 Olsson, 2010; 8 Sheidow et al., 2012; 9 Cary et al., 2013; 10 Dopp et al., 2014; 11. Borduin et al., 2015

^b^Scores were calculated by dividing the positively checked items on the quality checklist by the total minus items on the checklist that were not applicable (NA) to the study

### Risk of bias

All studies were RCTs [22–32]. Two of these studies [23, 32] only included patients receiving Medicaid (an aid program regarding insurances for low income families in the United States). For these studies, the RCT of the effect study contained (due to randomization) balanced samples. However, these samples were not checked for balance after the selection of participants who received medicaid, so they were at risk for selection bias. All studies had a high risk of performance bias, as blinding of both therapist and patient is impossible. For two studies [23, 32] blinding was not necessary as both the cost and outcome data were extracted from existing data systems (The medicaid billing records). Although blinding of outcome assessors is possible to reduce detection bias, no study reported to have done so. Blinding is also necessary for pre-allocation assessment. All studies were based on randomized controlled trials where allocation concealment is necessary. The studies included in this review, did not explicitly refer to the allocation concealment. Three studies were at risk of attrition bias. These three studies did not describe the number of patients that dropped out from the study [24, 27, 32]. Two studies only described the overall attrition rate [22, 25]. For one study [29] however, overall attrition rate could be extracted by using the study of French et al. [22] as it was based on the same participants. Dropout in the effect-study of Sheidow et al. [23] was low and although no dropout was described for the economic evaluation, as the economic evaluation is based on the same participants, this is expected to be low. Overall, dropout rate (when measured) seemed low. Reporting bias was assessed by reading protocols from the studies and no bias was reported. Only for two studies [22, 29] a protocol existed. Other studies did not have such a protocol, although for three studies trial registrations were present [24, 27, 28]. There were no indications of deviations from the original design. The economic evaluations did not always include all clinical outcomes that were available [23–26, 32] as there was often only interest in specific outcomes. One study [25] excluded clinical outcomes as there was no difference between treatments in terms of outcomes and so only costs were considered (costs minimization). The exclusion of outcomes was not related to possible negative impact on the results as effects in the studies were equally or more beneficial when compared to the effects of the comparator.

### Methodological summary

Uncertainty around treatment costs was not presented in four studies as averages of these costs were used [24, 27, 30, 31]. In six studies [22, 23, 25, 29–32] uncertainty around the (other) estimates was not (fully) addressed. In seven studies, a simple one way sensitivity analysis was used to assess the impact that changes in a certain parameter will have on the conclusions [22, 23, 26, 28, 30–32]. In two studies, sensitivity analysis was applied by imputing missing data in different ways. Outcomes proved to be robust [27, 28]. Two studies performed scenario analyses meaning that cost estimates (surrounded by uncertainty) were increased or decreased. Data proved to be robust [26, 32]. In another study a sensitivity analysis was carried out to assess the effect which outliers in each therapy group had on outcomes, but this did not have an effect the results. In the studies of Dopp et al. [31] and Borduin et al. [31] a sensitivity analysis was applied by using plausible minimum and maximum values (obtained from other studies) for offense categories, arrest rates and discount rates. French et al. [22] used different models which assessed the effect on using more or less covariates in the models but it did not affect the results. In six of the studies cost-effectiveness/utility/benefits were assessed based on models [22–25, 28, 32]. Four of these studies used simple regression models [23–25, 28] and two used a more advanced least squares random effect model [19, 26]. The remaining three studies did not integrate any model in the analysis. Three studies did not report their price year (the year to which costs are indexed) [23, 24, 32].

Authors of three studies indicated that a societal perspective was adopted, where not only health care costs but also other costs, for example those associated with lost or impaired ability to work, were taken into account [22, 26, 29]. However, this was only true for the study of Olsson [26], as this was the only study to assess costs outside the health care sector. In the studies of Dennis et al. [29] and French et al. [22], the societal part was defined as using market values for calculating the costs of goods and services used. Dopp et al. [30] and Borduin et al. [31] conducted cost-benefit analyses and did not explicitely mention their perspective. Both studies focused on taxpayer benefits and expressed intagible benefits in monetary values. Cary et al. [28] used a narrow perspective as only services that were recorded by a specific data-system were included (appointments with social workers, connexion workers (a United Kingdom (UK) governmental information, advice, guidance and support service for young people aged thirteen to nineteen), reparation workers (coordinates and supports a range of interventions and community reparation projects that young people will have to undertake as part of their Referral or Community Order), parenting workers, group workers and psychologists). Sheidow [23] adopted the perspective of an institution. Other studies did not explicitly state their perspective. Most of the studies only reported treatment costs. A summary of the costs and clinical outcomes measured in the studies is provided in Table 6.

**Table 6:** Overview of costs and clinical outcome measures used in studies

	Treatment costs	Other healthcare costs	Costs outside health care sector	Perspective used in the economic evaluations	Clinical outcome measure
(Schoenwald et al., 1996) [32]	✓	✓		Healthcare	-
(French et al., 2003) [22]	✓			Institution	-
(Sheidow et al., 2004) [23]	✓	✓		Healthcare	CBCL/GSI
(Dennis et al., 2004) [29]	✓			Institution	-
(McCollister et al., 2009) [24]	✓			Institution	SRD
(French et al., 2008) [25]	✓			Institution	YSR/days of marijuana use
(Olsson, 2010) [26]	✓		✓	Societal	-
(Sheidow et al., 2012) [27]	✓			Institution	TLFB/SRD
(Cary et al., 2013) [28]	✓			Institution	-
Dopp et al. (2014) [30]	✓		✓	Societal	-
Borduin et al. (2015) [31]	✓		✓	Societal	-

*CBCL* child behavior checklist, *GSI* global severity index, *SRD* self-report delinquency scale, *TLFB* timeline follow-back form, *YSR* youth self report

Following Drummond et al. [33], full economic evaluations should not only report costs, but also health outcomes. Four studies were classified as cost-effectiveness analyses [23, 25, 27, 29]. Only one of these studies compared treatments using an incremental cost-effectiveness ratio [29] as described for instance by Drummond et al. [33]. The cost-effectiveness analysis of French et al. [25] was reduced to a simple cost minimization analysis as the effects of both treatments after analysis proved to be similar. Sheidow et al. [27] calculated average cost-effectiveness ratios (ACER), which means that there was no direct comparison between treatments but only between the before- and after treatment costs and effects of every participant. In four studies it was explicitly stated that cost-benefit analyses [22, 26, 30, 31] were performed. Olsson [26] considered psychosocial and behavioral effects, but as no difference was observed regarding these clinical measures between treatments, these effects were excluded from the analysis. French et al. [22] did not value the health outcomes on which the intervention was focused (like reduction in days of substance use) but did value the effects of treatment on education, employment and criminal activity. Dopp et al. [30] and Borduin et al. [31] conducted a cost-benefit analysis; the cost outcome were the treatment costs and the benefits were defined as taypayer benefits, tangible benefits and intangible benefits were expressed in monetary values. Cary et al. [28] classified his study as a cost-offset evaluation. He calculated the net-benefit, but stated that his study cannot be viewed as a cost-effectiveness study as he did not measure health outcome. Two studies did not state the type of economic analyses they performed [24, 32], but did consider both costs and benefits. Mcollister [24] indicated that her study was not a full economic evaluation, as she only considered treatment costs. This is also the case concerning the study of Sheidow et al. [27], however, this study was stated to be a cost-effectiveness analysis. Furthermore, Schoenwald et al. [32] did not classify their study explicitly but considered both costs of different health care services and monetary benefits so it can be considered a cost-benefit analysis.

### Limitation/generalizability summary

Four studies commented on their generalizability [23, 25–27]. Sheidow et al. [23] reported that as their sample only consisted of youths enrolled in Medicaid, which are generally economically less advantaged, findings cannot be generalized to a more economically advantaged population. The same is true, although not stated, for the study of Schoenwald et al. [32] who also analyzed Medicaid data. The study of Olsson [26] was conducted in Sweden, where MST is twice more expensive than in the USA and may play a different role in society. MST in Sweden may be used as an alternative to nonplacement interventions as opposed to an alternative to placement interventions as found in other studies. Also in the study of French et al. [25], which was conducted in Mexico, location and small sample size were indicated as limitations for generalizability. The same was true, although not stated, for the study of Cary et al. [28] which was conducted in the United Kingdom. Also an important limitation (but not mentioned as such) were the omissions of uncertainty around the estimates in the studies of Dopp et al. [30] and Borduin et al. [31], so the results should be interpreted with caution. Furthermore, the study of Borduin et al. [31] was based on a very small (the smallest one in this review) sample size (only 48 patients) so uncertainty around the estimates (not reported) is expected to be high. Sensitivity analysis is not a solution for this problem as significance of the results cannot be determined (as the estimates in the sensitivity analysis are also subjected to uncertainty). The juvenile drug court programs, analyzed in the study of Sheidow et al. [27] are not easily generalized to other settings as they show great variation due to absence of a strict format. In addition, other settings may have different populations and salaries implying differences in costs. Almost all studies were cautious with drawing conclusions on their data. They not only recognized limitations within their research but also recognized that the number of economic evaluations is very limited and more research is needed before being able to draw conclusions [22–28, 32].

### Meta analysis

The data from the economic evaluations were not pooled as the population, setting, outcomes, costs and interventions were not comparable across studies.

## Discussion

This systematic review summarized and evaluated the cost-effectiveness of family/family-based therapy for adolescents with externalizing disorders, substance use disorder and delinquency. The overall quality of these studies was low, they produced mixed results.. Research should consider a wider perspective and take into account all relevant costs and effects using sophisticated models. Studies evaluating family/family-based therapy concerned various outcomes and costs, and investigated a variety of treatments in various populations in different settings. Therefore it was not possible to conduct a meta-analysis.

As expected, most of the studies were conducted in the United States where family/family-based treatments originate from [10, 11, 34]. The findings cannot be easily generalized to other health care systems as they differ between countries. The quality assessments showed that overall studies scored between 50 and 86 % and only two studies scored higher than 80 % [26, 28, 30, 31]. Studies that were conducted more recently, were in general higher of quality. When the two most recent studies [30, 31] were not considered, the quality of the studies overall was slightly higher for those studies originating from Europe. The quality of the two most recent studies was high when using the quality checklists, however, they also contained some important limitations. Firstly, although quality checklists only contain one question with respect to uncertainty around the estimates, it can be of paramount importance, especially when the sample size is low. Secondly, these studies are not easily generalized to an European setting as they conducted cost-benefit analyses, opposed to cost-effectiveness analyses that are commonly applied in European studies. Although the checklists used to assess quality of the studies depend on the subjective evaluation of the researchers and have yet not been validated, these two checklists have received much scrutiny and are therefore recommended [18]. Recommendations that follow from the quality assessment of the studies that were included in the review, are the following. Different treatments that are included in the study should be described more clearly so the differences and similarities between treatments are understandable. In many of the studies included in the review, the perspective taken was not mentioned or did not match with the categories of the costs that were included. In line with guidelines for economic evaluations the perspective should be stated [33]. A more broad perspective (societal versus healthcare) is recommended. The unit costs and resource use should be reported separately and a source of the references for the unit costs should be given. It is also important to explicitly mention whether a study is considered a cost-effectiveness/cost-benefit or cost-utility analysis.

Most studies included in the review used no model or simple models (regression). More complex models, like multilevel analysis, should be used. In this way covariates can be included, correlation between measurements over time can be addressed, missing data is accounted for and skewness in the costs and effects is considered. Uncertainty around costs should also be presented by using for instance bootstrapped costs/effects confidence intervals and can be visualized in a cost-effectiveness plane. Sensitivity analysis should be applied to variables that are uncertain (the rationale behind it should be explained). A one way sensitivity analysis is not always sufficient and a sensitivity anaysis also taking into account interactions between variables should be considered. A common discount rate should be applied for all costs and effects. Summary measures of the cost-benefit, cost-effectiveness or cost utility should be given. In case of a cost-effectiveness analysis incremental cost-effectiveness ratio (ICERS) should be calculated. For conducting economic evaluations it is advised to consult a health economist.

## Conclusions

Although family/family-based treatments are widely used and can be considered as effective for the treatment of a wide range of disorders [17], cost-effectiveness also needs to be addressed. Taking cost-effectiveness into account may have a large impact as family/family-based treatments are expensive. This review has summarized the economic evidence of family/family-based therapy for substance use disordersand delinquency in adolescents in a systematic and transparent way by using state of the art guidelines [18, 19]. As there are few studies evaluating the cost-effectiveness of family/family-based therapy and the quality of the existing studies is limited, new studies using higher quality standards are necessary. Large-scale implementation of these treatment models should be held back, until more evidence is available.

## Abbreviations

ACER, average cost-effectiveness ratio; ACRA, adolescent community reinforcement approach; AT, after treatment; C, comparator; CAU, care as usual; CBCL, child behavior checklist; CD, conduct disorder; CHEC, Consensus on Health Economic Criteria; CM, contingency management; DC, drug court; ENG, England; ERIC, Education Resource information Centre; FC, family court with community services; FFT, functional family therapy; FSN, family support network; Group, skill-focused psycho-education group intervention; GSI, global severity index; I, intervention; ICER, incremental cost-effectiveness ratio; IT, individual treatment; Joint, combination of individual and family therapy; MDFT, multidimensional family therapy; MeSH, Medical Subject Headings; MET/CBT12, motivational enhancement treatment/cognitive behavior therapy, 12 sessions; MET/CBT5, motivational enhancement treatment/cognitive behavior therapy, 5 sessions; MEX, Mexico v; MST, multisystemic therapy; MST-PSB, multisystemic therapy for problem sexual behavior; NA, not applicable; NC, not clear; NHS EED, NHS Economic Evaluation Database; NS, not stated; NS1, reference to non-accessible article; PC, psychiatric crisis; PRISMA, Preferred Reporting for Systematic reviews and Meta-Analyses; RCT, randomized controlled trial; SD, standard deviation; SRD, self-report delinquency scale; SUD, substance use disorder; SW, Sweden; TLFB, timeline follow-back form; UK, United Kingdom; USA, United States of America; USD, United States Dollar; YSR, youth self report

## Appendix 1

**Table 7:** Description family/family-based interventions

Family/family-based interventions	
MST	Target family interaction and the extended social systems in youths with substance abuse problems, delinquency or antisocial behavior / Permits separate meetings adolescent but preference for family /More focus on antisocial behavior/ focused both on family functioning and on extra familial functioning / Treatment team not actively involved as observers and actors but team is only self-reflexive/ Treatment team actively involved as observers and actors /degree of severity higher and combination of more problems
FFT	Target family interaction and the extended social systems in youths with substance abuse problems, delinquency or antisocial behavior/ Almost no separate meetings adolescent /More focus on antisocial behavior/More focused on family functioning less on extra familial functioning/ Treatment team not actively involved as observers and actors but team is only self-reflexive/ explicitly emphasizes therapist is integral part of the system/degree of severity lower
MDFT	Target family interaction and the extended social systems in youths with substance abuse problems, delinquency or antisocial behavior/ Separate meetings adolescent/ Focus on substance abuse / focused both on family functioning and on extra familial functioning /Treatment team not actively involved as observers and actors but team is only self-reflexive/degree of severity higher

Sources: Leukehof et al. and Oudhof et al. (Leukefeld et al., 2008; Oudhof et al., 2009)

*MST* multisystemic therapy, *FFT* functional family therapy, *MDFT* multidimensional family treatment

## Appendix 2

**Search terms Pubmed**

1. “family therapy”[MESH]
2. “Functional family therapy”
3. (FFT NOT (“fast Fourier transform” OR “freedom-from-transfusion” OR “fast Fourier transforms” OR “fast Fourier transformation” OR “Far-Field Transform”))
4. “Multisystemic Therapy”
5. (MST NOT (“microbial source tracking” OR “minimum spanning tree”))
6. “Multidimensional Treatment Foster Care”
7. “MTFC”
8. “multidimensional family therapy”
9. “MDFT”
10. “family behavior therapy”
11. “FBT”
12. brief strategic family therapy”
13. “BSFT”
14. “family based therapy”[Title/Abstract]
15. “family based interventions”[Title/Abstract]
16. “family based intervention”[Title/Abstract]
17. “family systems intervention” [Title/Abstract]
18. “family systems interventions” [Title/Abstract]
19. “family system intervention” [Title/Abstract]
20. “family system interventions” [Title/Abstract]
21. “family intervention program”[Title/Abstract]
22. “family intervention programs”[Title/Abstract]
23. “systemic Therapy” [Title/Abstract]
24. OR 1–23
25. “economic evaluation” [title/Abstract]
26. “economic evaluations” [title/Abstract]
27. “cost effective” [title/Abstract]
28. “cost effectiveness” [title/Abstract]
29. “cost utility analysis” [title/Abstract]
30. “costs” [Title/Abstract] AND “effect”[Title/Abstract]
31. “cost” [Title/Abstract] AND “effect”[Title/Abstract]
32. “cost” [Title/Abstract] AND “effects”[Title/Abstract]
33. “costs” [Title/Abstract] AND “effects”[Title/Abstract]
34. “costs”[Title/Abstract] AND “benefits”[Title/Abstract]
35. “cost” [Title/Abstract] AND “benefit”[Title/Abstract]
36. “costs” [Title/Abstract] AND “benefit”[Title/Abstract]
37. “cost” [Title/Abstract] AND “benefits”[Title/Abstract]
38. “costs” [Title/Abstract] AND “utility”[Title/Abstract])
39. “cost” [Title/Abstract] AND “utility”[Title/Abstract])
40. “cost” [Title/Abstract] AND “utilities”[Title/Abstract]
41. “costs” [Title/Abstract] AND “utilities”[Title/Abstract])
42. “Cost Analysis” [title/Abstract]
43. “Cost Measures” [title/Abstract]
44. “cost benefit analysis”[title/Abstract]
45. “cost measure” [title/Abstract]
46. “cost” [title]
47. “costs” [title]
48. “cost benefit analysis” [MESH]
49. OR 25–48
50. NOT (cancer[Title/Abstract]OR psoriasis[Title/Abstract]OR “radiation therapy”[Title/Abstract] OR diabetes[Title/Abstract] OR diabetic[Title/Abstract] OR obesity [Title/Abstract] OR aids[Title/Abstract] OR HIV[Title/Abstract] OR sarcomas[Title/Abstract] OR chemotherapy[title/Abstract]))
51. 24 AND 49 AND 50

**Search terms Eric, Psycinfo and Cochrane**

In Eric, the same search terms were used except for the MESH terms. In psycinfo, the MESH terms were replaced with APA’s thesaurus of Psychological index Terms and in cochrane, the same terms were used.
